# Investigation of the direct and indirect mechanisms of primary blast insult to the brain

**DOI:** 10.1038/s41598-021-95003-9

**Published:** 2021-08-06

**Authors:** Jose E. Rubio, Ginu Unnikrishnan, Venkata Siva Sai Sujith Sajja, Stephen Van Albert, Franco Rossetti, Maciej Skotak, Eren Alay, Aravind Sundaramurthy, Dhananjay Radhakrishnan Subramaniam, Joseph B. Long, Namas Chandra, Jaques Reifman

**Affiliations:** 1 Department of Defense Biotechnology High Performance Computing Software Applications Institute, Telemedicine and Advanced Technology Research Center, United States Army Medical Research and Development Command, ATTN: FCMR-TT, 504 Scott Street, Fort Detrick, MD 21702-5012 USA; 2 The Henry M. Jackson Foundation for the Advancement of Military Medicine, Inc., 6720A Rockledge Drive, Bethesda, MD 20817 USA; 3 Blast Induced Neurotrauma Division, Center for Military Psychiatry and Neurosciences, Walter Reed Army Institute of Research, 503 Robert Grant Drive, Silver Spring, MD 20910 USA; 4 Department of Biomedical Engineering, Center for Injury Biomechanics, Materials, and Medicine, New Jersey Institute of Technology, 111 Lock Street, Newark, NJ 07103 USA

**Keywords:** Neuroscience, Brain injuries, Brain injuries

## Abstract

The interaction of explosion-induced blast waves with the head (i.e., a direct mechanism) or with the torso (i.e., an indirect mechanism) presumably causes traumatic brain injury. However, the understanding of the potential role of each mechanism in causing this injury is still limited. To address this knowledge gap, we characterized the changes in the brain tissue of rats resulting from the direct and indirect mechanisms at 24 h following blast exposure. To this end, we conducted separate blast-wave exposures on rats in a shock tube at an incident overpressure of 130 kPa, while using whole-body, head-only, and torso-only configurations to delineate each mechanism. Then, we performed histopathological (silver staining) and immunohistochemical (GFAP, Iba-1, and NeuN staining) analyses to evaluate brain-tissue changes resulting from each mechanism. Compared to controls, our results showed no significant changes in torso-only-exposed rats. In contrast, we observed significant changes in whole-body-exposed (GFAP and silver staining) and head-only-exposed rats (silver staining). In addition, our analyses showed that a head-only exposure causes changes similar to those observed for a whole-body exposure, provided the exposure conditions are similar. In conclusion, our results suggest that the direct mechanism is the major contributor to blast-induced changes in brain tissues.

## Introduction

The detonation of an explosive device can potentially cause a wide spectrum of injuries to the brain. These injuries may include those presumably caused by the explosion-induced blast overpressure (BOP) interacting directly with the body and the head (i.e., non-impact, primary-blast injury), as well as secondary and tertiary injuries caused by propelled fragments and the acceleration of the body, respectively^1^. Unlike secondary and tertiary injuries, there is still no consensus on whether or how the exposure to a BOP causes non-impact, primary traumatic brain injury (TBI)^2^.

Several competing hypotheses, broadly grouped into those involving a *direct* or an *indirect* mechanism, propose potential pathways by which a BOP could cause primary TBI. Hypotheses based on a direct mechanism assume that injury to the brain results from the interaction of the BOP with the head, including skull deformation^3^, head acceleration^4^, cavitation^5,6^, and high-pressure differentials in brain tissues derived from the propagation of the wave through the skull and brain^7–9^. In contrast, hypotheses based on an indirect mechanism postulate that injury to the brain involves the interaction of the BOP with the body, wherein the BOP compresses the abdomen and chest, and transfers its kinetic energy to the body’s internal organs, including the brain and the blood as fluid medium^1,10^.

Due to the limited understanding of how the direct and indirect mechanisms could cause blast-induced TBI, studies that characterize the relative contributions of each of these pathways to brain-tissue changes are necessary to address this knowledge gap. To this end, a few studies investigated the effects of the direct and indirect mechanisms on the brain tissues by exposing animals to a blast wave while attempting to separately shield their head or their torso^10,11^. However, because a shield cannot properly isolate a body region placed inside the shock tube from the incident wave^12^, it is possible that their results^10,11^ may not adequately represent the individual contributions of each mechanism to blast-induced brain changes.

We can delineate more effectively each mechanism by considering configurations wherein we isolate a specific body region from the blast wave by keeping it outside of the shock tube^12^. Indeed, a few studies characterized molecular changes in brain tissues by subjecting rats to a head-only exposure with the torso outside of the shock tube^13–15^. In addition, our group previously conducted torso-only exposures on rats (with the animal’s head secured outside of the shock tube) to investigate the wave-body interaction and implement computational models that characterize the biomechanical responses on the tissues and vessels of the brain due to the indirect mechanism^12^. While these studies helped advance our knowledge of how the wave-body interaction could injure the brain, the potential role of each mechanism in causing blast-induced TBI remains largely undefined.

In the present study, we conducted separate shock-tube experiments on rats to isolate the potential direct and indirect mechanisms of primary-blast injury, and characterized the relative contributions of each to the observed brain-tissue changes. To assess the direct mechanism, we subjected rats to either a whole-body or a head-only blast exposure. To evaluate the indirect mechanism, we subjected rats to either a whole-body or a torso-only blast exposure. We then performed histopathological and immunohistochemical analyses on coronal brain slices collected from control and blast-exposed rats to identify global and regional brain-tissue changes due to each mechanism. We hypothesized that we could characterize the effects of the direct and indirect mechanisms of primary-blast injury on the brain using animal experiments designed to isolate each of the mechanisms.

## Materials and methods

To characterize brain-tissue changes in rats resulting from the direct and indirect mechanisms, we performed separate shock-tube experiments at an incident BOP of 130 kPa on 10- to 12-week-old (330–350 g) male Sprague–Dawley rats (Charles River Laboratories). The Animal Care and Use Review Office of the U.S. Army Medical Research and Development Command, Fort Detrick, MD, as well as the Institutional Animal Care and Use Committees at the Walter Reed Army Institute of Research (WRAIR), Silver Spring, MD, and the New Jersey Institute of Technology (NJIT), Newark, NJ, approved all experimental protocols. We conducted all experiments in compliance with the Animal Research: Reporting of In Vivo Experiments (ARRIVE) guidelines.

### Shock-tube experimental setups

#### Direct mechanism

To investigate changes in brain tissues resulting from the direct mechanism, we considered a whole-body configuration, wherein we exposed the head and torso of the rat to the blast wave, and a head-only configuration, in which we isolated the torso from blast exposure by keeping it outside of the shock tube. To enhance the blast-wave interaction with the body of the animal, we performed all experiments with the animal positioned in a vertical orientation facing the incident blast wave. We previously used these configurations to investigate the influence of animal orientation on the biomechanical responses of the brain when exposed to a blast wave^16^.

We used an Advanced Blast Simulator (ABS)^17^ located at WRAIR to perform the direct-mechanism experiments. The compressed-air ABS consisted of 152- and 6,400-mm-long driver and driven sections, respectively, separated by Valmex membranes. The driver section had a divergent cross-sectional area, and the driven section had a square cross-sectional area of 610 mm × 610 mm. The driven section extended 1,067 mm into an end-wave eliminator, which mitigated the pressure waves that could reflect back into the test section of the ABS (i.e., the secondary pressure waves) and the residual gas. The ABS generated an incident wave with positive- and negative-pressure phases. We positioned the animal at a distance of 2825 mm from the membranes.

**Figure 1:**
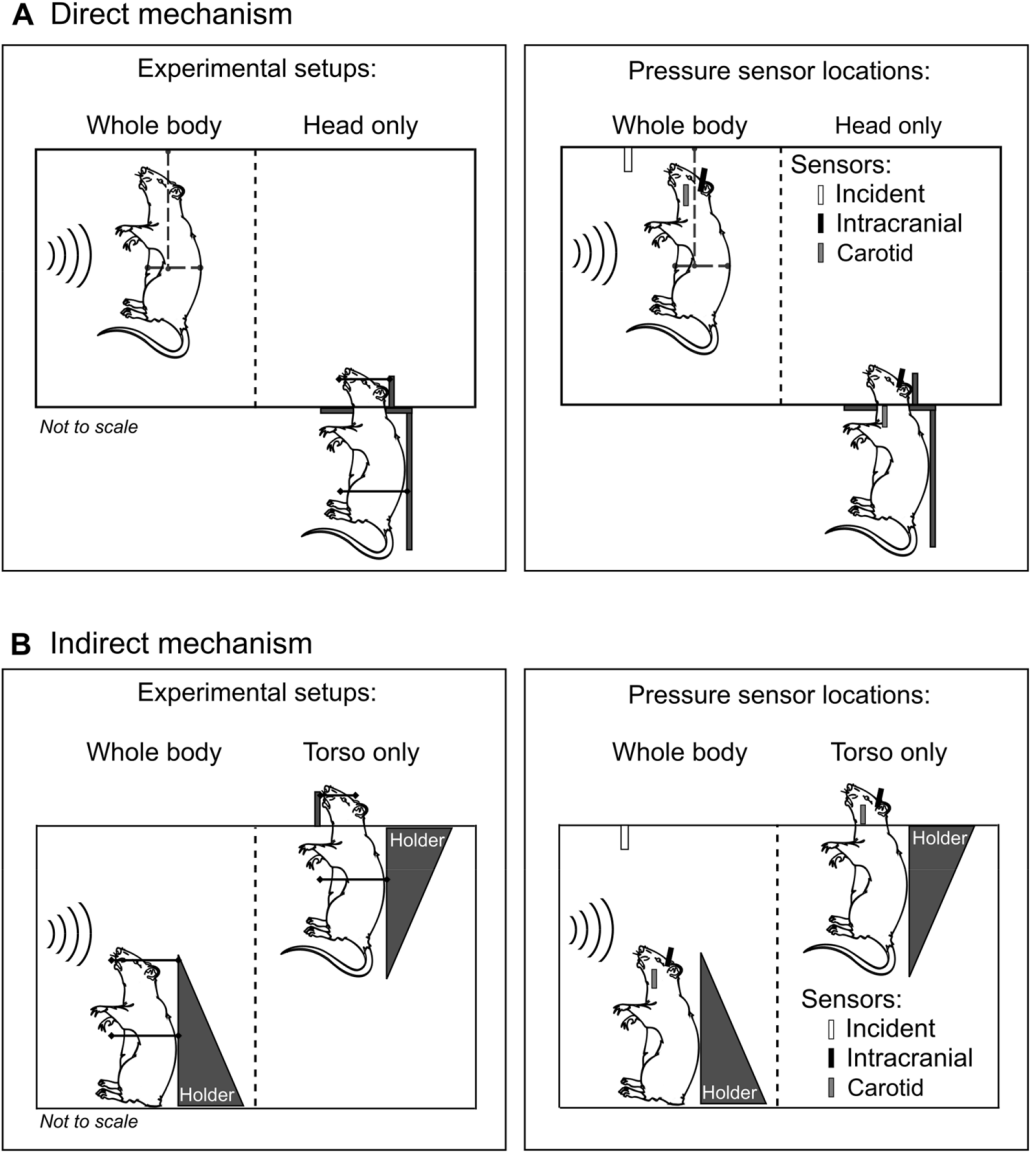
Shock-tube experiments to isolate two potential mechanisms of primary-blast injury. (**A**) We considered whole-body and head-only blast-exposure configurations to investigate the effects of the direct mechanism. In the whole-body configuration setup, we mounted the rat in a custom-made sling inside the test section of the shock tube. This setup minimally restrained the animal’s motion. In the head-only configuration setup, we isolated the torso of the rat from the blast exposure by securing it to a vertical cylinder outside of the shock tube and allowed the head to protrude into the test section through a small opening. Additionally, we used flexible strings wrapped around the head to keep it in a vertical position without restraining its motion. We measured the incident, intracranial, and carotid-artery pressures at the locations shown by the white, black, and gray vertical bars, respectively, in the schematic on the right (we omitted the restraining strings to better illustrate the locations of the sensors). (**B**) We considered whole-body and torso-only blast-exposure configurations to investigate the effects of the indirect mechanism. In the whole-body configuration setup, we mounted the rat in a holder inside the test section of the shock tube, and used a thin cotton cloth and Velcro straps to secure the animal and minimize its motion. In the torso-only configuration setup, we isolated the head of the rat from the blast exposure by keeping it outside of the shock tube and securing the torso to a holder inside the test section. We measured the incident, intracranial, and carotid-artery pressures at the locations shown in the schematic on the right (we omitted the restraining Velcro straps to better illustrate the locations of the sensors).

In the whole-body configuration setup to assess the direct mechanism, we minimally restrained the animal in a custom-made sling suspended from the overhead rails inside the test section of the shock tube (Fig. 1A, left). In contrast, in the head-only configuration setup, we secured the torso of the rat to a vertical silicone cylinder outside of the shock tube, and arranged for the head to project into the test section through an opening in the bottom wall (Fig. 1A, left). Additionally, we wrapped flexible strings (1 to 2 mm thick) around the head and attached them to two vertical pins to keep the head in a vertical orientation without impeding its motion.

#### Indirect mechanism

We investigated changes in brain tissues caused by the indirect mechanism using a whole-body configuration, in which we exposed the head and torso of the rat to the blast wave, and a torso-only configuration, wherein we limited the blast exposure exclusively to the torso by keeping the head outside of the shock tube. We previously used the torso-only configuration to characterize the role of the indirect mechanism on the biomechanical responses of the brain vessels and tissues^12^.

We used two rectangular shock tubes located at the NJIT to conduct these experiments^18,19^. For the whole-body exposures, we used a compressed-gas shock tube consisting of 438- and 6,871-mm-long driver and driven sections, respectively, separated by Mylar membranes. This shock tube had a cylindrical driver section with an inner diameter of 197 mm. The driven section had a square cross-sectional area of 711 mm × 711 mm, where we positioned the animal at a distance of 3310 mm from the membranes. For the torso-only exposures, we used a compressed-gas shock tube consisting of 552- and 6,000-mm-long driver and driven sections, respectively, separated by Mylar membranes. The driver section had a circular cross-sectional area with an inner diameter of 197 mm. The driven section had a square cross-sectional area of 228 mm × 228 mm, where we positioned the animal at a distance of 3080 mm from the membranes. Both shock tubes at the NJIT had an end-plate to mitigate the secondary pressure waves. For the whole-body exposures, we used the larger shock tube to avoid potential interactions between the animal and pressure waves reflected off the shock-tube walls.

In the whole-body configuration setup to assess the indirect mechanism, we secured the head and torso of the animal to a holder inside the test section of the shock tube using a thin cotton cloth and Velcro straps (Fig. 1B, left). In the torso-only configuration setup, we arranged the head of the rat to protrude out of the test section through an opening in the upper wall. We then restrained the head using Velcro straps against a vertical fixture, with the torso kept within the test section, mounted on a holder, and secured by a thin cotton cloth and Velcro straps (Fig. 1B, left). In both setups, we tightly secured the head and torso of the rat to minimize the motion of the animal during the blast exposures.

### Pressure measurements

#### Direct mechanism

We randomly assigned rats to whole-body or head-only configuration groups (n = 5 each). We anesthetized the animals using isoflurane and exposed them to a single blast using their respective setups in the ABS at WRAIR. For each experiment, we measured the static pressure–time profile of the blast wave at a distance of 2825 mm from the membranes using a custom-made pencil probe (Stumptown Research and Development, Marion, NC) equipped with a pressure sensor (model 8515C-50; Meggitt Sensing Systems, Irvine, CA), with its sensing surface parallel to the flow of the blast wave. Additionally, we measured the intracranial pressure at the lateral ventricle and the intravascular pressure at the carotid artery, using Millar pressure catheters (model SPR-407; ADInstruments, Colorado Springs, CO). In the head-only configuration setup, the carotid-artery pressure sensor was located outside of the shock tube (Fig. 1A, right). To implant the sensors, we followed the protocol described in our previous work^16^. We connected all pressure sensors to a data recorder (model TMX-18; Astro-Nova, Inc., West Warwick, RI) and sampled all measurements at a frequency of 0.8 MHz.

#### Indirect mechanism

We arbitrarily assigned rats to whole-body or torso-only configuration groups (n = 8 each). We used isoflurane to anesthetize the animals. Subsequently, we exposed them to a single blast using their respective setups and shock tubes at the NJIT. We measured the static pressure–time profile of the blast wave at distances of 2946 and 2692 mm from the membranes for whole-body and torso-only exposures, respectively, using a pressure sensor (model 134A24; PCB Piezotronics, Depew, NY) with its probe oriented parallel to the flow of the blast wave. We measured the intracranial pressure at the lateral ventricle and the intravascular pressure at the carotid artery using Millar pressure catheters (models SPR-407 and SPR-671, respectively, ADInstruments). In the torso-only configuration setup, the intracranial and carotid-artery pressure sensors were located outside of the shock tube (Fig. 1B, right). We implanted the sensors following the approach described in our previous study^12^. For data acquisition, we used a custom LabVIEW code running on a multifunction data acquisition module (model PXI-6133; National Instruments, Austin, TX) and a PXI chassis (model PXIe-1082; National Instruments). We recorded the data at a sampling frequency of 1.0 MHz.

### Assessment of changes in brain tissues

Separate from the group of animals used for pressure measurements, we used additional groups to assess changes in brain tissues due to blast exposure. Based on a power calculation using Chow’s method^20^, we determined that a sample size of n = 4 was sufficient to observe a statistical difference (p < 0.05) between groups with a statistical power of 0.80. Hence, for the direct-mechanism study, we randomly assigned rats to control (n = 4), whole-body (n = 10), or head-only (n = 10) configuration groups, whereas for the indirect-mechanism study, we randomly assigned rats to control, whole-body, or torso-only configuration groups (n = 4 each). We did not implant any pressure sensors in these animals. All blast-exposed animals (i.e., whole-body, head-only, and torso-only groups) were anesthetized using isoflurane and received a single blast using their respective experimental setups and shock tubes. Control animals received the same treatment, except for the exposure to the blast wave. To identify changes in brain tissues at 24 h after blast exposure, we conducted histopathological and immunohistochemical analyses on 12 coronal brain slices (30-µm thick), which were serially cut from − 1 to − 12 mm relative to Bregma.

#### Tissue collection

Twenty-four hours after blast exposure, we anesthetized the rats with isoflurane and transcardially perfused them with phosphate-buffered saline (PBS), followed by 4% paraformaldehyde acid (PFA) to fix the brains. We extracted the brains from the cranial vaults and incubated them in 4% PFA for 48 h. We sent all fixed brains for all configurations to FD Neurotechnologies (Columbia, MD) for histopathological and immunohistochemical staining.

#### Histopathology

We conducted histopathological analyses on the coronal brain slices, using silver staining, to elucidate signs of neuronal degeneration as indicated by axonal fiber-tract deterioration due to blast exposure.

At FD Neurotechnologies, the fixed brains were cryopreserved by immersion in 30% sucrose solution. Subsequently, they were embedded in optimal cutting temperature media, frozen in isopentane pre-cooled to − 70 °C, and stored at − 80 °C. Each frozen brain was initially cut into 500-µm-thick coronal sections. Next, from each section, 14 slices were cut and collected in PBS, wherein the first four slices had a thickness of 50 µm and the remaining 10 slices had a thickness of 30 µm. From each set of 14 slices, the sixth slice (30-µm thick) was processed using FD NeuroSilver Kit II (#PK301; FD Neurotechnologies) to detect neuronal degeneration. After the staining procedure, the coronal slices were shipped back to their respective laboratories of origin (WRAIR or NJIT) for quantification analyses.

#### Immunohistochemistry

We conducted immunohistochemical analyses on the coronal brain slices to assess the expressions of glial fibrillary acidic protein (GFAP), ionized calcium-binding adapter molecule 1 protein (Iba-1), and neuron-specific nuclear protein (NeuN) after blast exposure. The expressions of GFAP, Iba-1, and NeuN are associated with the distributions of astrocytes, microglia, and neuronal cells, respectively^21^.

At FD Neurotechnologies, the brain slices were incubated with primary antibodies to detect GFAP (#556330; BD Biosciences, San Jose, CA; 1:350 dilution), Iba-1 (#019-19741; Wako Chemicals, Richmond, VA; 1:350 dilution), and NeuN (#ABN90P; Millipore Sigma, Burlington, MA; 1:250 dilution). This was followed by incubation in PBS containing the following secondary reporter antibodies with a fluorescent tag: Alexa Fluor 488 donkey anti-mouse (#A21202; Thermo Fisher Scientific, Waltham, MA; 1:250 dilution), Alexa Fluor 594 donkey anti-rabbit (#A21207; Thermo Fisher Scientific; 1:250 dilution), and Alexa Fluor 405 streptavidin conjugate (#S32351; Thermo Fisher Scientific; 1:200 dilution) for the detection of GFAP, Iba-1, and NeuN primary antibodies, respectively. The stained coronal slices were shipped back to their respective laboratories of origin (WRAIR or NJIT) for quantification analyses.

#### Quantification

**Figure 2:**
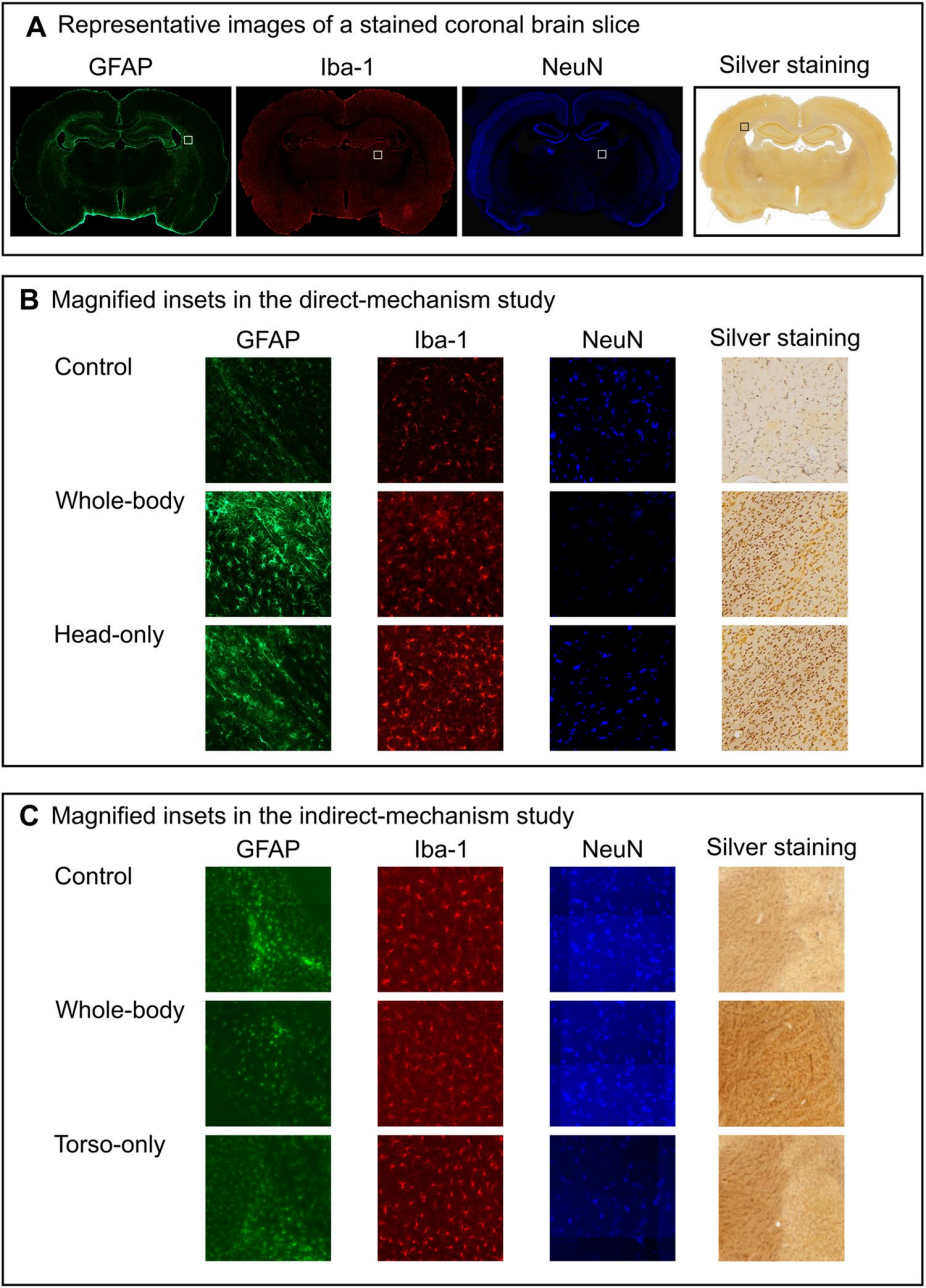
Representative images of immunohistochemical- and histopathological-stained coronal brain slices. In both the direct- and indirect-mechanism studies, we conducted immunohistochemical analyses on the coronal brain slices harvested from control and blast-exposed rats to assess the distribution of astrocytes, microglia, and neuronal cells using GFAP, Iba-1, and NeuN staining, respectively. In addition, we conducted histopathological analyses using silver staining to assess for neuronal degeneration. After staining, we digitized each coronal brain slice with a 10-× magnification using bright-field for histopathological analysis and fluorescence filters for immunohistochemical analyses. (**A**) Representative images of each staining assessment in a coronal brain slice located at − 2 mm relative to Bregma. (**B**,**C**) The magnified insets show examples of stained cells in coronal brain slices of control and blast-exposed rats (**B**) in the direct-mechanism study and (**C**) in the indirect-mechanism study. Note that the images in panel (**C**) representative of the indirect mechanism are not illustrated here in high resolution because the acquisition software in this study did not allow us to export the high-resolution images used in the analysis.

We digitized each coronal brain slice harvested from rats in the direct-mechanism study (i.e., control, whole-body, and head-only groups) with a 10-× magnification using a photomicroscope (model BX-61; Olympus America, Inc., Center Valley, PA). In addition, we used bright-field and fluorescence (i.e., green for GFAP, red for Iba-1, and blue for NeuN) filters to acquire the brain-slice images for the histopathological and immunohistochemical analyses, respectively. Figure 2A shows representative images of each staining assessment in a coronal brain slice. For each coronal slice and filter, we then used the ImagePro software (Media Cybernetics, Rockville, MD) to quantify the intensity (i.e., the integrated density).

We digitized each coronal brain slice harvested from rats in the indirect-mechanism study (i.e., control, whole-body, and torso-only groups) with a 10-× magnification using an Aperio Versa 200 digital pathology scanner (Leica Biosystems, Wetzlar, Germany). We used the same filters described above in the direct-mechanism study to acquire the images. For the histopathological analyses, we quantified the number of positively stained cells that showed signs of axonal degeneration within each slice using the Aperio Image Scope software (Leica Biosystems). For the immunohistochemical analyses, we quantified the intensity for each fluorescence filter and coronal slice using the Aperio Area-Quantification FL algorithm (Leica Biosystems). Because of the proprietary format of the images acquired in the indirect-mechanism study, we were not able to conduct the analyses using the same software and quantification algorithm employed in the direct-mechanism study.

### Statistical analysis

We conducted whole-brain analyses to identify overall changes in brain tissues between control and blast-exposed groups. For these analyses, we implemented a linear mixed-effects model using the statistical software R and the package lme4^22,23^. In this model, we considered the group (i.e., control, whole-body, head-only, and torso-only) as a factor and assigned a random intercept to the brain-tissue change measure (i.e., the intensity of the silver precipitates or the number of positively stained cells for the histopathological analyses, and the fluorescence intensity for the immunohistochemical analyses) to account for the within-animal dependence. To determine the statistical significance of a given change in brain tissue, we used a likelihood-ratio test that compared the linear mixed-effects model with the factor to a model without it (i.e., a null model). We then performed Dunnett’s post hoc test on the linear mixed-effects model for pairwise comparisons between the control and blast-exposed groups, using the Holm–Bonferroni correction to account for multiple comparisons. Additionally, we performed regional analyses where we compared the data across each coronal slice using the Kruskal–Wallis test. We tested for differences between blast-exposed groups and their respective controls using Dunnett’s post hoc test with the Holm–Bonferroni correction. We set the significance criterion for both whole-brain and regional analyses to p < 0.05.

### Disclaimer

The opinions and assertions contained herein are the private views of the authors and are not to be construed as official or as reflecting the views of the United States (U.S.) Army, the U.S. Department of Defense, or The Henry M. Jackson Foundation for the Advancement of Military Medicine, Inc. This paper has been approved for public release with unlimited distribution.

### Ethics statement

In conducting research using animals, the investigator(s) adhered to the Animal Welfare Act Regulations and other Federal statutes relating to animals and experiments involving animals and the principles set forth in the current version of the Guide for Care and Use of Laboratory Animals, National Research Council.

## Results

### Pressure measurements

#### Direct mechanism

**Figure 3:**
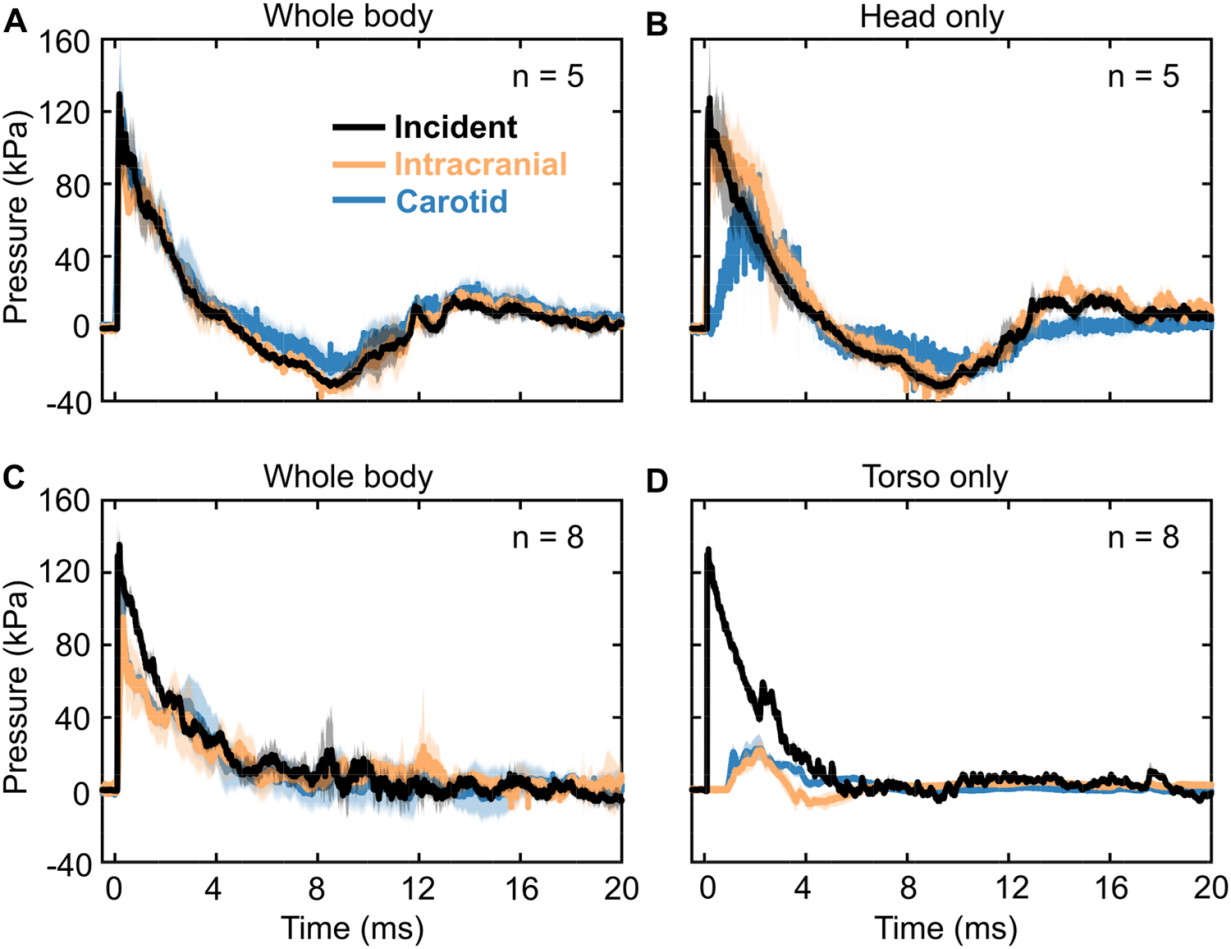
Pressure–time profiles of the incident, intracranial, and carotid-artery pressures for the (**A**) whole-body and (**B**) head-only blast-exposure experiments in the direct-mechanism study, and the (**C**) whole-body and (**D**) torso-only blast-exposure experiments in the indirect-mechanism study. The solid lines and shaded areas represent the mean and one standard deviation, respectively.

The three pressure–time profiles (i.e., incident, intracranial, and carotid artery) from the whole-body blast-exposure experiments in the direct-mechanism study (Fig. 3A) showed a nearly instantaneous rise to the peak overpressure of 130 kPa, followed by a rapid nonlinear decay that transitioned into a negative-pressure phase, and a subsequent return to the baseline condition. In addition, the incident and intracranial pressures had similar peak pressures, durations, and impulses in both phases (Table 1). In contrast, the carotid-artery pressure showed a slightly higher peak overpressure and impulse relative to the incident pressure in the positive-pressure phase (Table 1).

**Table 1:** Summary of relevant blast-wave parameters (mean ± one standard deviation) measured in the shock-tube experiments.

Measurement	Parameter					
	Positive phase			Negative phase		
	Peak overpressure (kPa)	Impulse (kPa∙ms)	Duration (ms)	Peak underpressure (kPa)	Impulse (kPa∙ms)	Duration (ms)
**Direct mechanism: Whole body (n = 5)**						
Incident	130.2 ± 16.9	192.9 ± 35.9	4.6 ± 0.2	− 33.1 ± 2.2	− 117.0 ± 21.1	6.9 ± 0.2
Intracranial	130.2 ± 8.1	186.5 ± 21.9	4.3 ± 0.8	− 40.1 ± 7.2	− 123.4 ± 33.4	6.9 ± 0.5
Carotid	155.4 ± 35.6	218.5 ± 50.8	5.2 ± 0.7	− 32.5 ± 5.9	− 61.1 ± 18.8	5.4 ± 0.4
**Direct mechanism: Head only (n = 5)**						
Incident	127.7 ± 19.5	220.2 ± 55.1	4.8 ± 0.3	− 33.5 ± 4.4	− 134.7 ± 28.1	7.3 ± 0.3
Intracranial	118.5 ± 16.6	268.9 ± 56.2	4.6 ± 0.3	− 47.9 ± 3.2	− 125.2 ± 15.7	7.9 ± 0.4
Carotid	67.1 ± 7.0	149.5 ± 23.3	4.4 ± 0.3	− 35.9 ± 10.6	− 96.6 ± 25.9	8.2 ± 0.7
**Indirect mechanism: Whole body (n = 8)**						
Incident	133.1 ± 13.7	282.0 ± 34.6	6.9 ± 1.4	–	–	–
Intracranial	114.8 ± 23.0	202.5 ± 75.3	5.9 ± 1.6	–	–	–
Carotid	132.3 ± 16.1	230.3 ± 61.6	5.8 ± 1.3	–	–	–
**Indirect mechanism: Torso only (n = 8)**						
Incident	133.2 ± 5.1	236.2 ± 12.6	5.2 ± 0.1	–	–	–
Intracranial	24.3 ± 6.6	26.4 ± 4.6	2.4 ± 0.2	–	–	–
Carotid	28.9 ± 5.5	55.9 ± 12.5	5.1 ± 1.6	–	–	–

The incident and intracranial pressure–time profiles from the head-only blast-exposure experiments (Fig. 3B) showed trends similar to those described above for the whole-body experiments. In contrast, the carotid-artery pressure showed a slower rise to a lower peak overpressure, although it decayed in the manner described above for the whole-body experiments. The magnitudes of the intracranial and carotid-artery peak overpressures were lower than the incident peak overpressure (Table 1).

#### Indirect mechanism

All pressure–time profiles (i.e., incident, intracranial, and carotid artery) from the whole-body blast-exposure experiments in the indirect-mechanism study (Fig. 3C) rose instantaneously to the peak overpressure and decayed rapidly and nonlinearly, without a negative-pressure phase. Compared to the incident peak overpressure, the magnitude of the intracranial peak overpressure was lower, whereas that of the carotid-artery pressure was similar to the incident overpressure (Table 1).

For the torso-only blast-exposure experiments (Fig. 3D), only the incident pressure showed a significant peak overpressure, followed by a rapid nonlinear decay. In contrast, the intracranial and carotid-artery pressures rose to considerably lower peak values (Table 1 and Fig. 3D).

### Whole-brain analyses

#### Direct mechanism

**Figure 4:**
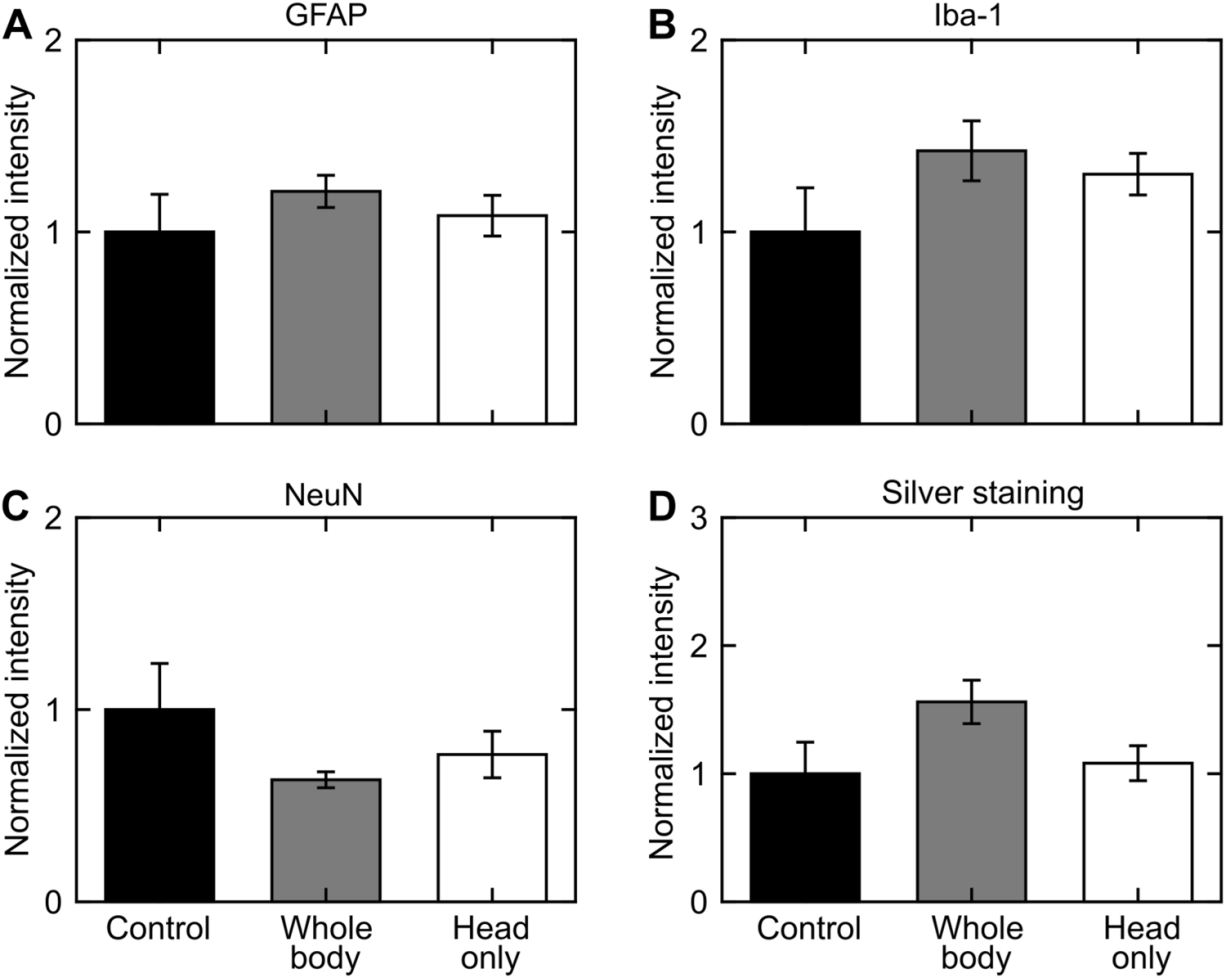
Changes in the rat brain due to whole-body or head-only blast exposure in the direct-mechanism study, as indicated by (**A**) GFAP-positive, (**B**) Iba-1-positive, (**C**) NeuN-positive, and (**D**) silver staining. Asterisks denote statistically significant differences (p < 0.05) between blast-exposed (n = 10) and control (n = 4) groups. The bar heights and vertical line lengths represent the mean and one standard error of the mean (SEM), respectively. We determined per-animal estimates by averaging the values of the 12 coronal brain slices for each animal. For each group, we then determined the mean and SEM using the respective per-animal estimates. Lastly, for each staining assessment, we normalized the data from the blast-exposed groups (i.e., whole-body and head-only) by the data from their respective controls. We normalized the data for presentation purposes only. We conducted all statistical analyses using the raw data with their respective values. We did not find a statistically significant difference in any of the staining assessments between control and blast-exposed rats.

Albeit not statistically significant when compared to controls, for the direct mechanism, GFAP-positive (Fig. 4A), Iba-1-positive (Fig. 4B), and silver-positive staining increased in whole-body- and head-only-exposed rats (Fig. 4D). In contrast, relative to controls, NeuN-positive staining decreased in whole-body- and head-only-exposed rats (Fig. 4C).

#### Indirect mechanism

**Figure 5:**
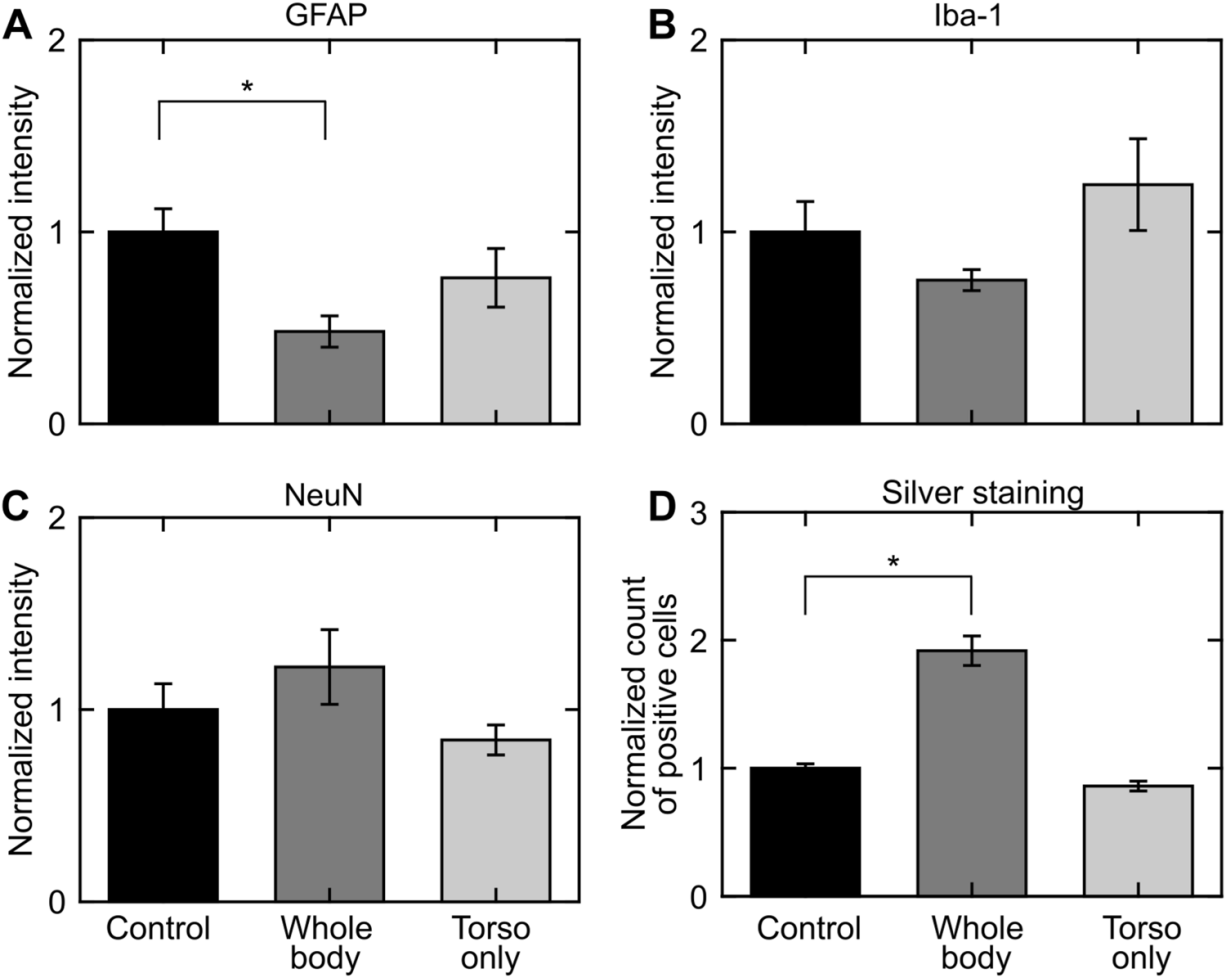
Changes in the rat brain due to whole-body or torso-only blast exposure in the indirect-mechanism study, as indicated by (**A**) GFAP-positive, (**B**) Iba-1-positive, (**C**) NeuN-positive, and (**D**) silver staining. Asterisks denote statistically significant differences (p < 0.05) between blast-exposed (n = 4) and control (n = 4) groups. The bar heights and vertical line lengths represent the mean and one standard error of the mean (SEM), respectively. We determined per-animal estimates by averaging the values of the 12 coronal brain slices for each animal. For each group, we then determined the mean and SEM using the respective per-animal estimates. Lastly, for each staining assessment, we normalized the data from the blast-exposed groups (i.e., whole-body and torso-only) by the data from their respective controls. We normalized the data for presentation purposes only. We conducted all statistical analyses using the raw data with their respective values.

For the indirect mechanism, relative to controls, GFAP-positive staining decreased (p < 0.05) in whole-body-exposed rats but not in torso-only-exposed rats (Fig. 5A). Whole-brain analyses revealed no statistically significant changes in Iba-1-positive and NeuN-positive staining (Fig. 5B,C, respectively) for whole-body- and torso-only-exposed rats when compared to controls. Additionally, silver staining showed that relative to controls, the number of positively stained cells increased (p < 0.05) in whole-body-exposed rats but not in torso-only-exposed rats (Fig. 5D).

### Regional analyses

#### Trends in controls

**Figure 6:**
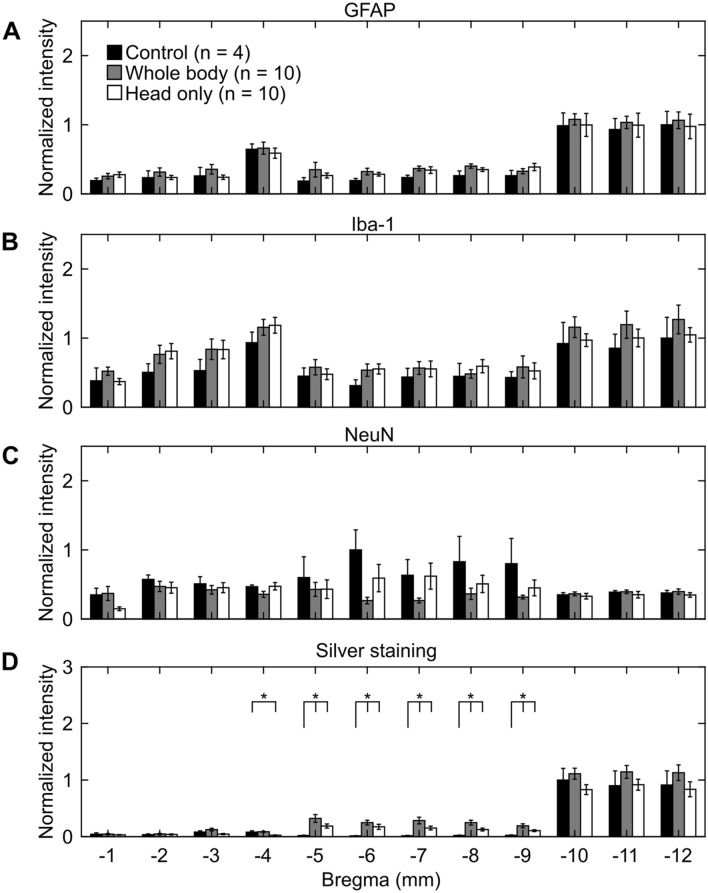
Changes in coronal brain slices due to whole-body or head-only blast exposure in the direct-mechanism study, as indicated by (**A**) GFAP-positive, (**B**) Iba-1-positive, (**C**) NeuN-positive, and (**D**) silver staining. Asterisks denote statistically significant differences (p < 0.05) between blast-exposed (n = 10) and control (n = 4) groups. The bar heights and vertical line lengths represent the mean and one standard error of the mean (SEM), respectively. At each brain-slice region for each group, we determined the mean and SEM using the corresponding brain-slice data. For each staining assessment, we then normalized the data for controls by the highest control value. Lastly, we normalized the data from blast-exposed groups (i.e., whole-body and head-only) by the data from their respective controls at each region. We normalized the data for presentation purposes only. We conducted all statistical analyses using the raw data with their respective values.

**Figure 7:**
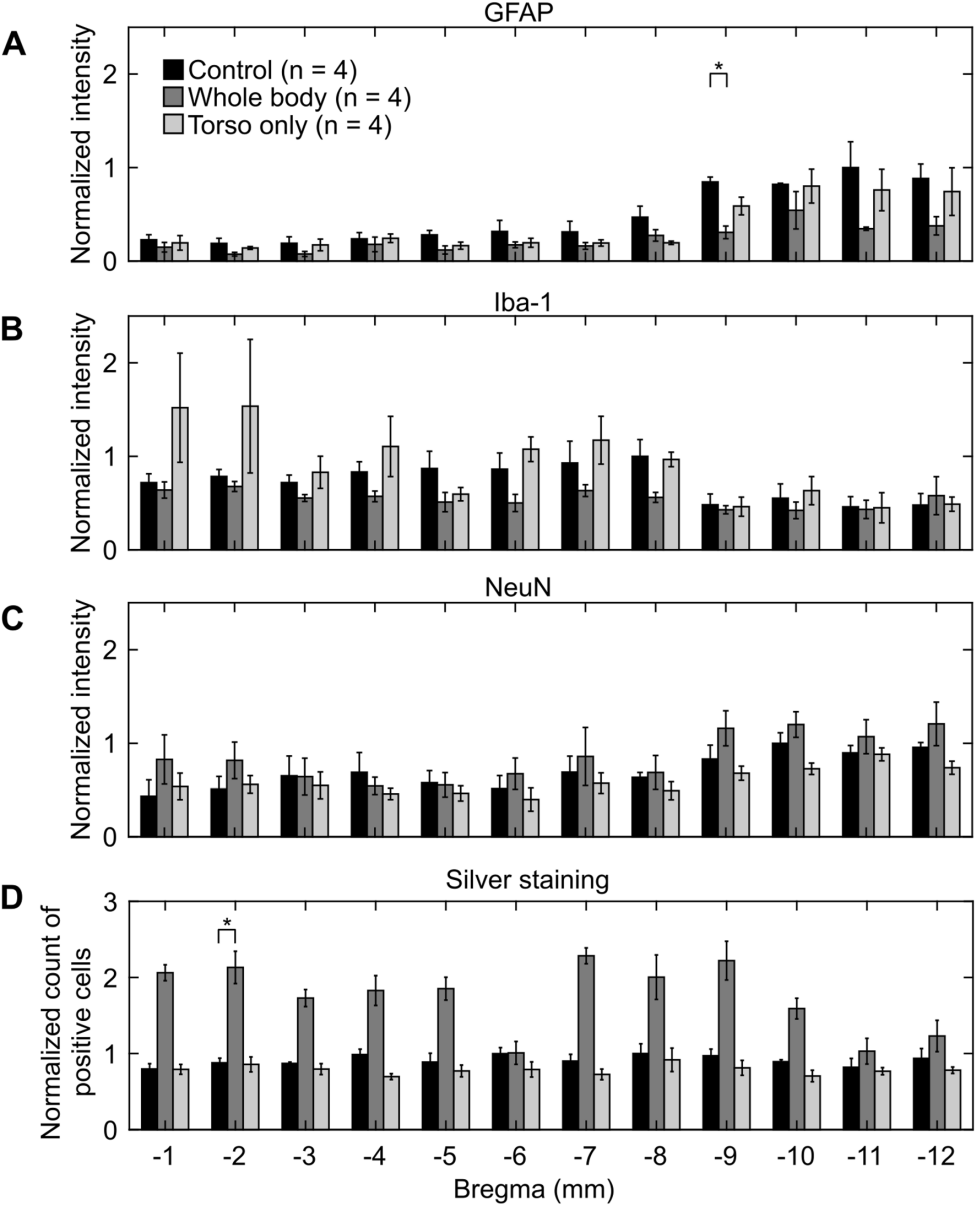
Changes in coronal brain slices due to whole-body or torso-only blast exposure in the indirect-mechanism study, as indicated by (**A**) GFAP-positive, (**B**) Iba-1-positive, (**C**) NeuN-positive, and (**D**) silver staining. Asterisks denote statistically significant differences (p < 0.05) between blast-exposed (n = 4) and control (n = 4) groups. The bar heights and vertical line lengths represent the mean and one standard error of the mean (SEM), respectively. At each brain-slice region for each group, we determined the mean and SEM using the corresponding brain-slice data. For each staining assessment, we then normalized the data for controls by the highest control value. Lastly, we normalized the data from blast-exposed groups (i.e., whole-body and torso-only) by the data from their respective controls at each region. We normalized the data for presentation purposes only. We conducted all statistical analyses using the raw data with their respective values.

We observed regional variations in the positive-staining results for control rats in both studies (Figs. 6 and 7). When compared across the different brain regions, GFAP-positive staining was higher in the posterior region of the brain (from − 9 or − 10 to − 12 mm relative to Bregma) for controls in both studies (Figs. 6A and 7A). Similarly, when compared with other brain regions, Iba-1-positive staining was higher in the posterior region (from − 10 to − 12 mm relative to Bregma) for controls in the direct-mechanism study (Fig. 6B), whereas it was lower in a similar region (from − 9 to − 12 mm relative to Bregma) for controls in the indirect-mechanism study (Fig. 7B). In addition, NeuN-positive staining was higher in the middle region of the brain (from − 5 to − 9 mm relative to Bregma) for controls in the direct-mechanism study (Fig. 6C), whereas it was higher in the posterior region of the brain (from − 9 to − 12 mm relative to Bregma) for controls in the indirect-mechanism study (Fig. 7C). Lastly, silver-positive staining was higher in the posterior region of the brain (from − 10 to − 12 mm relative to Bregma) for controls in the direct-mechanism study (Fig. 6D), whereas the controls in the indirect-mechanism study did not show an evident regional variation (Fig. 7D).

#### Direct mechanism

Although not statistically significant when compared to controls, the regional analyses revealed that GFAP-positive and Iba-1-positive staining increased in most of the brain regions for whole-body- and head-only-exposed rats (Fig. 6A,B, respectively). When compared to controls, whole-body- and head-only-exposed rats showed lower levels of NeuN-positive staining in the anterior and middle regions of the brain (from − 1 to − 9 mm relative to Bregma; Fig. 6C). Lastly, our regional analyses showed that relative to controls, silver-positive staining increased (p < 0.05) in head-only-exposed rats (from − 4 to − 9 mm relative to Bregma; Fig. 6D) and whole-body-exposed rats (from − 5 to − 9 mm relative to Bregma; Fig. 6D).

#### Indirect mechanism

Relative to controls, GFAP-positive staining decreased consistently throughout the brain (p < 0.05 at − 9 mm relative to Bregma) for whole-body-exposed rats (Fig. 7A). Similarly, our analyses showed that GFAP-positive staining decreased in torso-only-exposed rats (Fig. 7A), although not to the extent that we observed in whole-body-exposed rats. Albeit not statistically significant, Iba-1-positive staining decreased and NeuN-positive staining increased marginally in most of the brain regions for whole-body-exposed rats when compared to controls (Fig. 7B,C, respectively). In contrast, relative to controls, a torso-only exposure caused a marginal increase in Iba-1-positive staining and a modest decrease in NeuN-positive-staining (Fig. 7B,C, respectively). Regional analyses of silver staining revealed that relative to controls, the number of positively stained cells increased throughout the brain for whole-body-exposed rats with a statistically significant increase at − 2 mm relative to Bregma (Fig. 7D). In contrast, we observed a slight decrease in the torso-only exposure.

### Brain-structure analyses

We conducted additional brain-structure analyses wherein we evaluated changes in GFAP and Iba-1 in the brainstem and cerebellum of head-only- and whole-body-exposed rats in the direct-mechanism study. For this purpose, we delineated the brainstem and cerebellum in each of the slices from − 10 to − 12 mm relative to Bregma. Then, we quantified the corresponding intensity for each slice and averaged the results separately for GFAP and Iba-1. When compared to controls, we did not find any statistically significant changes in GFAP or Iba-1 levels in the brainstem or the cerebellum of the blast-exposed rats (Supplementary Fig. 1). These results are consistent with those from the whole-brain and regional analyses (Figs. 4A,B, 6A,B), which indicate that the whole-brain and regional analyses adequately describe the changes resulting from the direct and indirect mechanisms.

## Discussion

The contributions, if any, of the direct and indirect mechanisms to non-impact, primary TBI remain uncertain. To address this knowledge gap, we systematically conducted shock-tube experiments on rats. Using head-only, torso-only, or whole-body blast-exposure configurations, we exposed rats to an incident BOP of 130 kPa and measured the incident, intracranial, and carotid-artery pressures to characterize the wave-body interaction due to each mechanism. Then, we conducted histopathological and immunohistochemical analyses on brains harvested from control and blast-exposed rats at 24 h following blast exposure to evaluate the independent contribution of each mechanism to blast-induced changes in the brain tissues.

In our experimental configurations, we isolated either the torso or the head of the rat from the blast wave by placing it outside of the shock tube. In our previous study^12^, we demonstrated that keeping the head outside of the tube is more effective in isolating it from the pressure wave than using head shields made of different materials. Based on those results, to study the indirect mechanism, we considered a torso-only configuration, wherein we positioned the head of the animal outside of the shock tube and exposed only the torso to the blast wave (Fig. 1B). In contrast, to study the direct mechanism, we implemented a head-only configuration, in which we exposed only the head of the animal by keeping the torso outside of the tube (Fig. 1A).

For the head-only exposures, the pressure wave propagated through the rat’s head with a comparable magnitude to the incident pressure. Our results indicated that the intracranial peak overpressure was 93% of the incident pressure (Table 1 and Fig. 3B). This was similar to the results for the whole-body exposure, where we observed a negligible difference in peak overpressures and temporal profiles between the incident and intracranial pressures (Table 1 and Fig. 3A).

The pressure measurements at the carotid artery (outside of the shock tube) from the head-only exposures suggested that the torso was largely isolated in this configuration. Relative to the incident pressure, the peak overpressure at the carotid artery decreased by 48% for this exposure (Table 1 and Fig. 3B). As expected, these measurements were markedly different from those for the whole-body exposure, where the carotid-artery peak overpressure was 19% higher than the incident pressure (Table 1 and Fig. 3A).

The intracranial and carotid-artery pressure measurements in the torso-only exposures provided evidence that the head was largely isolated in this configuration (Table 1 and Fig. 3D). By obtaining measurements from pressure sensors placed outside of the shock tube, we determined that the intracranial and carotid-artery peak overpressures in the torso-only exposures were 82% and 78% lower, respectively, than the incident peak pressure (Table 1 and Fig. 3D). In contrast, when the entire body of the rat was exposed to the blast wave (i.e., whole-body exposure), the intracranial and carotid-artery peak overpressures were 86% and 99% of the incident peak pressure, respectively (Table 1 and Fig. 3C).

We assessed the relative contribution of each mechanism to changes in the brain tissues by comparing the histopathological and immunohistochemical results for head-only- and torso-only-exposed rats with their respective whole-body-exposed groups. In the direct-mechanism study, relative to controls, head-only- and whole-body-exposed rats showed similar trends in all analyses (Figs. 4 and 6), which suggests that a head-only exposure, in the absence of thoracic exposure, causes brain-tissue changes comparable to those induced by a whole-body exposure. In contrast, in the indirect-mechanism study, relative to controls, torso-only- and whole-body-exposed rats showed opposite trends in all analyses, except for GFAP (Figs. 5 and 7).

Despite using standardized experimental protocols in the two studies, for the coronal brain slices, we noticed an inter-study variability in the trends of controls in all analyses, except for GFAP (Figs. 6 and 7). For GFAP, we consistently observed the highest levels in the posterior region of the brain (from –10 to –12 mm relative to Bregma; Figs. 6A and 7A). In contrast, for the remaining analyses (Figs. 6B–D and 7B–D), the trends in the controls were inconsistent when compared across studies, possibly because of confounding factors associated with the handling of animals and laboratory environments. While using a different technique to evaluate brain-tissue changes due to blast exposure, Kawa et al. also found a discrepancy between control animals from two different laboratories and attributed these differences to psychological stressors derived from laboratory environments^24^. In fact, Kamnaksh et al. demonstrated that animal handling and laboratory conditions induce psychological stressors that trigger molecular changes in the brain of rats with no history of blast exposure^25^. In the present study, to minimize the potential effects of confounding factors, each laboratory used their own group of control animals to investigate a specific mechanism.

In the direct-mechanism study, relative to controls, the whole-body and head-only exposures caused a modest increase in GFAP (Figs. 4A and 6A). Consistently, previous studies using similar blast-exposure configurations have also reported elevated GFAP levels in the rat brain at 24 h and 7 days after exposure^13,14,26–28^. In our study, the increase in GFAP could be attributed to astrocyte reactivity (Fig. 2B) in response to neuroinflammation^29–31^. Indeed, multiple studies support the notion that inflammation persists in the rat brain at 24 h following blast exposure^32,33^. Therefore, our results likely reflect an early regulatory response of astrocytes against inflammation.

Relative to controls, the levels of Iba-1 increased in whole-body- and head-only-exposed rats in the direct-mechanism study, suggesting a mild activation of microglia due to the exposure (Figs. 2B, 4B and 6B). Similar to reactive astrocytes, the activated microglia facilitate the neuroinflammatory response and tissue regeneration following an insult to the brain^34^. Consistent with our results, other studies also reported evidence of activated microglia in the rat brain at 24 h after blast exposure^32,35^.

The trend in NeuN (Figs. 4C and 6C) for whole-body- and head-only-exposed rats in the direct-mechanism study indicates a minor loss of neurons due to blast exposure, predominantly in the middle region of the brain (from − 5 to − 9 mm relative to Bregma, Fig. 6C). Using a whole-body configuration with the rat positioned in a prone orientation, Cho et al. observed trends in Iba-1 and NeuN that were similar to those in our study at multiple time points (ranging from 4 h to 2 weeks) following blast exposure^32^. In addition, using a different assessment (i.e., Fluoro-Jade staining), other investigations observed neurodegeneration in the rat brain at different time points (ranging from 3 to 72 h) following a whole-body^26,36^ and a head-only exposure^15^.

Our analyses in the direct-mechanism study revealed higher levels of silver staining (Figs. 4D and 6D) in whole-body- and head-only-exposed rats relative to controls. These results suggest neuronal and axonal fiber-tract deterioration at 24 h after blast exposure (Fig. 2B), especially for the region ranging from − 4 to − 9 mm relative to Bregma (p < 0.05). Our histopathological finding is consistent with previous studies in rats, which reported neuronal and axonal damage in major white-matter tracts at 24 h and up to 7 days after a single whole-body exposure^37–39^.

In the indirect-mechanism study, relative to controls, a whole-body exposure caused a significant change in GFAP (p < 0.05), while a torso-only exposure caused a modest change (Fig. 5A). When compared to controls, whole-body-exposed rats showed lower GFAP levels, especially in the posterior region of the brain (from − 9 to − 12 mm relative to Bregma; Figs. 5A and 7A). Interestingly, this downward trend was the opposite of the trend observed for whole-body-exposed rats in the direct-mechanism study (Figs. 4A and 6A). The decreased GFAP levels due to a whole-body exposure in the indirect-mechanism study could be associated with the death of astrocytes. Indeed, one study reported the death of brain cells in rats at different time points (ranging from 3 to 72 h) after a whole-body exposure of 117 kPa^26^. However, their report did not distinguish between astrocytes, microglia, and other brain cells. While using an open-field blast-exposure configuration, Pun et al. observed DNA-fragmented astrocytes in the rat brain at 24 h after blast exposure^40^. Using a similar configuration but with non-human primates, Lu et al. observed a structural alteration and apoptosis of astrocytes in the brain of these animals for up to one month after blast exposure and suggested that these alterations could compromise the regulatory functions of astrocytes^41^. Lastly, Miller et al. observed the demise of astrocytes using an in vitro model of blast-induced injury^42^.

The torso-only exposure caused a modest decrease in GFAP (Figs. 5A and 7A). While using a whole-body-exposure configuration, Gama Sosa et al. also observed a GFAP reduction in whole-brain homogenates and isolated cerebral vessels in rats exposed to multiple blasts (evaluated 6 weeks after the last exposure)^43^. They associated the decrease in GFAP with structural alterations in the walls of cerebral vessels (i.e., a degeneration of astrocytic end-feet attached to the cerebral vasculature). However, because their observations are based on a whole-body-exposure configuration, it is unknown which mechanism caused the vascular changes. Our results and the results from Gama Sosa et al. suggest a potential contribution of the indirect mechanism to changes in the walls of the cerebral vessels. Similarly, our previous study showed that a torso-only exposure can generate a blood surge to the brain, considerably increasing the wall shear stress on the cerebral vessels, and supporting the plausibility of vascular injury due to the indirect mechanism^12^.

In the indirect-mechanism study, relative to controls, the whole-body exposure caused a modest decrease in Iba-1, while the torso-only exposure caused a slight increase (Figs. 5B and 7B). In whole-body-exposed rats, the decrease in Iba-1 occurred predominantly in the middle region of the brain and could be associated with the death of microglia (from − 3 to − 8 mm relative to Bregma; Fig. 7B). In contrast, the slight increase in Iba-1 in torso-only-exposed rats corresponded to isolated microglia (Fig. 2C). However, these cells did not show substantially proliferated processes nor were they clustered, which are characteristics of activated microglia^34^.

Relative to controls, our analyses in the indirect-mechanism study revealed a slight increase in NeuN in whole-body-exposed rats and a small decrease in torso-only-exposed rats (Figs. 5C and 7C). Albeit not statistically significant, the increase in NeuN in whole-body-exposed rats was unexpected. In contrast, the decrease in NeuN in torso-only-exposed rats could be explained by a minor loss of neurons resulting from the blast exposure.

Compared to controls, we observed a higher count of positively silver-stained cells (p < 0.05, Fig. 5D) for whole-body-exposed rats in the indirect-mechanism study, but not for torso-only-exposed rats. In addition, for the whole-body exposures, the results for silver staining agreed in the direct- and indirect-mechanism studies. However, instead of being predominantly higher in the middle region of the brain (i.e., from – 5 to – 9 mm relative to Bregma; Fig. 6D), the positively stained cells for whole-body-exposed rats in the indirect-mechanism study were distributed homogeneously across the brain (Fig. 7D).

Interestingly, when comparing results across studies, we noted that the whole-body exposures caused brain-tissue changes in opposite directions. For example, relative to their respective controls, the whole-body-exposed rats showed GFAP levels that were higher in the direct-mechanism study (Figs. 4A and 6A) but lower in the indirect-mechanism study (Figs. 5A and 7A). We also observed this discrepancy for Iba-1 and NeuN. The discrepancies may be attributed to one or a combination of the following factors: the different software systems used to quantify the fluorescence intensity, a difference in shock-tube setup, the negative-pressure phase in the experiments of the direct-mechanism study, or the different apparatus used to constrain head motion during the blast exposure. In addition, other potential sources of discrepancy include differences in animals from various batches or differences in the handling of animals.

We believe that the apparatus used to constrain head motion is the most likely source of the differences in the whole-body results between the two studies. Using a head-only side-on blast-exposure configuration, Sawyer et al. observed a small reduction in GFAP levels in several regions of the rat brain when head movement was minimized^13^. In contrast, they observed a significant elevation in GFAP levels in the same brain regions when the head of the rat was less constrained. To some extent, the degree of head movement allowed by the restraints in the Sawyer et al. study was comparable to that allowed in our study. Based on a qualitative analysis of high-speed video footage acquired during our experiments, we observed that the body and the head of the rats in our direct-mechanism study displaced in a rigid-motion manner along the direction of the shock front. In contrast, the body and head of the rats in the indirect-mechanism study showed reduced motion during blast exposure. Consistent with Sawyer et al., our results show that GFAP levels in the brain are lower when head motion is minimized.

Our studies have limitations. First, to evaluate the brain-tissue changes resulting from each mechanism, the studies had small sample sizes. However, based on a power analysis, we determined that a sample size of n = 4 for each group was sufficient to observe a statistical difference in protein expression in brain tissues between control and blast-exposed groups. Indeed, using this sample size, we found significant changes in GFAP and silver staining in whole-body-exposed rats in the indirect-mechanism study (p < 0.05; Figs. 5A and 7A). Second, we conducted experiments at two separate laboratories, which had the potential to confound our results due to inter-laboratory differences. However, to minimize such a possibility, we uniformized most study protocols between laboratories (e.g., we sent samples for histopathological staining to the same vendor), made sure that each laboratory had their own group of controls and whole-body-exposed rats, and analyzed the data by comparing each laboratory’s results against their own controls and whole-body-exposed rats. Third, our findings are based on one histopathological (i.e., silver staining) and three immunohistochemical analyses (i.e., GFAP, Iba-1, and NeuN staining). We selected these analyses because they allowed for the evaluation of different brain cells (i.e., neurons, astrocytes, and microglia) that are known to be affected by blast exposure^26,32,44,45^. Nonetheless, we acknowledge that analyses based on other proteins or techniques may reveal additional contributions to changes in the brain tissues. Lastly, we only investigated the acute effects of blast (i.e., at 24 h after exposure). Future studies should further characterize the mid- and long-term effects, if any, of each mechanism on brain tissues.

To conclude, we separately characterized the changes in the rat brain due to the direct and indirect mechanisms of potential primary-blast injury. To this end, we used head-only, torso-only, and whole-body configurations to expose separate groups of rats to an incident BOP of 130 kPa. To characterize the wave-body interaction due to each mechanism, we measured the incident, intracranial, and carotid-artery pressures. For the head-only and whole-body exposures, our measurements showed a negligible difference in peak overpressures and temporal profiles between the incident and intracranial pressures, suggesting a similar loading to the head resulting from these two configurations. In contrast, for the torso-only exposure, the peak intracranial and carotid-artery pressures were substantially lower than the incident peak pressure, which provides evidence that the head was largely isolated from the incident wave in this configuration. Then, we conducted histopathological and immunohistochemical analyses to characterize the changes in the brain tissues at 24 h after blast exposure. Our results indicated that a whole-body exposure could cause bidirectional changes in GFAP and Iba-1 levels in brain tissues, possibly because of the presence of a negative-pressure phase in the incident blast wave or differences in the degree of head-motion constraint during the blast exposures. These bidirectional changes suggest that a whole-body exposure could potentially induce glial (i.e., astrocytes and microglia) activation or cause cellular death at 24 h after exposure, depending on the exposure condition. Our results also showed that the head-only exposure, in the absence of thoracic exposure, resulted in brain-tissue changes similar to those observed for whole-body exposure when using comparable exposure conditions. Additionally, a torso-only exposure, in the absence of head exposure, caused no appreciable brain-tissue changes identifiable by histopathological (using silver staining) or immunohistochemical analyses (based on GFAP, Iba-1, and NeuN). In summary, our work suggests that the direct mechanism, rather than the indirect mechanism, is the major contributor to changes in the rat brain 24 h after primary blast exposure.

## Supplementary Information

Supplementary Information.

## Data Availability

All data will be made available following a written request to the corresponding author, along with a summary of the planned research.
