# Supplementary material for: Investigation of the direct and indirect mechanisms of primary blast insult to the brain
Source: Sci Rep. 2021 Aug 6;11:16040. doi: 10.1038/s41598-021-95003-9 (PMC8346555; doi:10.1038/s41598-021-95003-9)
Supplement: Supplementary file 1 — Supplementary Information. [file 41598_2021_95003_MOESM1_ESM.pdf]

# Investigation of the Direct and Indirect Mechanisms of Primary Blast Insult to the Brain

**Jose E. Rubio[1,2], Ginu Unnikrishnan[1,2], Venkata Siva Sai Sujith Sajja[3], Stephen Van Albert[3], Franco Rossetti[3], Maciej Skotak[3,4], Eren Alay[4], Aravind Sundaramurthy[1,2], Dhananjay Radhakrishnan Subramaniam[1,2], Joseph B. Long[3], Namas Chandra[4], and Jaques Reifman[1]**

[1]Department of Defense Biotechnology High Performance Computing Software Applications Institute, Telemedicine and Advanced Technology Research Center, United States Army Medical Research and Development Command, Fort Detrick, MD 21702, USA

[2]The Henry M. Jackson Foundation for the Advancement of Military Medicine, Inc., 6720A Rockledge Drive, Bethesda, MD 20817, USA

[3]Blast Induced Neurotrauma Division, Center for Military Psychiatry and Neurosciences, Walter Reed Army Institute of Research, 503 Robert Grant Drive, Silver Spring, MD 20910, USA

[4]Department of Biomedical Engineering, Center for Injury Biomechanics, Materials, and Medicine, New Jersey Institute of Technology, 111 Lock Street, Newark, NJ 07103, USA

## Supplementary Material

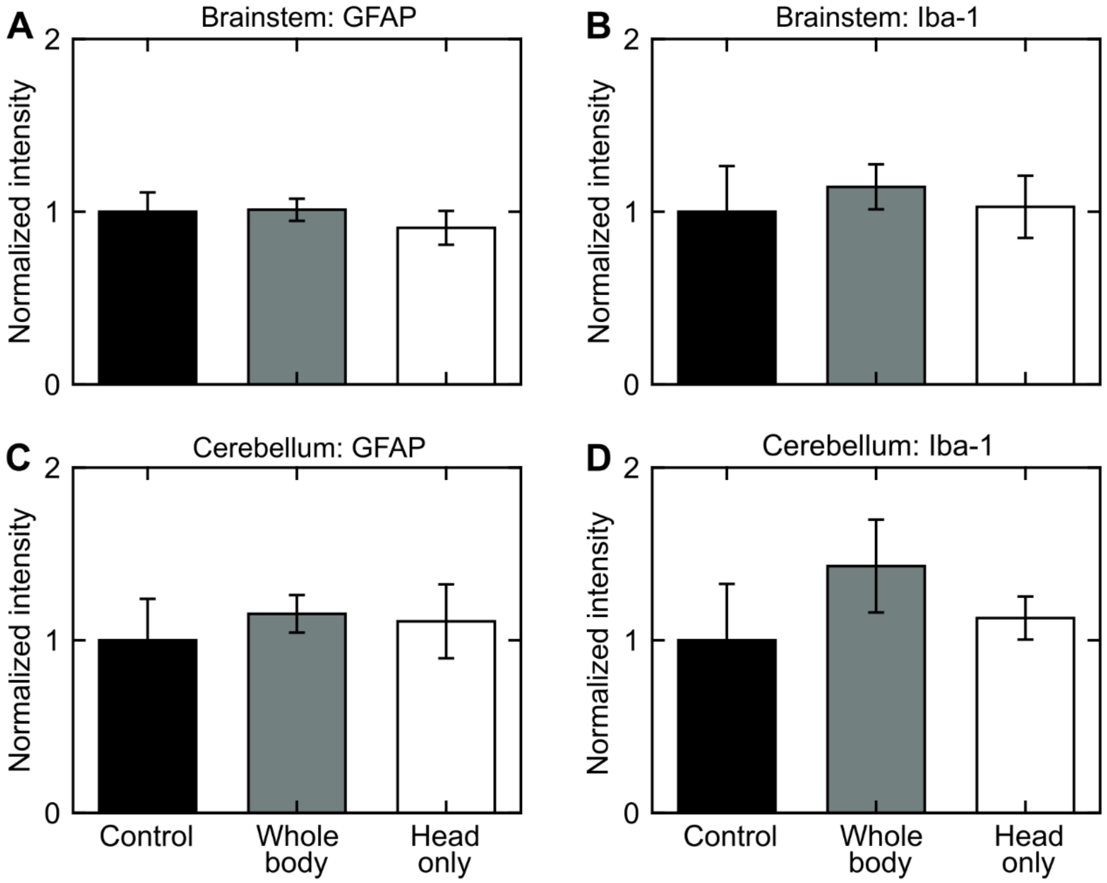


**Supplementary Figure 1.** Changes in the brainstem and cerebellum of a rat due to whole-body or head-only blast exposures in the direct-mechanism study, as indicated by (A and C) GFAP-positive and (B and D) Iba-1-positive staining. We implemented a linear mixed-effect model to identify changes between control (n = 4) and blast-exposed groups (n = 10 each). The bar height and vertical line length represent the mean and one standard error of the mean (SEM), respectively. We delineated the brainstem and cerebellum in each of the slices from −10 to −12 mm relative to Bregma. Next, we determined per-animal estimates by averaging the values of the three coronal slices for each animal. For each group, we then determined the mean and SEM using the respective per-animal estimates. Lastly, for each staining assessment, we normalized the data from the blast-exposed groups (i.e., whole-body and head-only) by the data from their respective controls. We normalized the data for presentation purposes only. We conducted all statistical analyses using the raw data with their respective values. When compared to controls, we did not find any statistically significant changes in GFAP- or Iba-1-positive staining in the brainstem or the cerebellum of head-only- and whole-body-exposed rats. These results are consistent with those from the whole-brain and regional analyses in the accompanying manuscript (Figs. 4A, 4B, 6A, and 6B), which indicate that such analyses adequately describe the changes resulting from the direct and indirect mechanisms.
